# Chitosan nanoparticle applications in dentistry: a sustainable biopolymer

**DOI:** 10.3389/fchem.2024.1362482

**Published:** 2024-04-10

**Authors:** Roma Mascarenhas, Shreya Hegde, Nidhi Manaktala

**Affiliations:** ^1^ Department of Conservative Dentistry and Endodontics, Manipal College of Dental Sciences Mangalore, Manipal Academy of Higher Education, Manipal, India; ^2^ Department of Oral Pathology and Microbiology, Manipal College of Dental Sciences Mangalore, Manipal Academy of Higher Education, Manipal, India

**Keywords:** chitosan, sustainable nanomaterials, organic-inorganic hybrid nanomaterial, bio-medical applications, dentistry

## Abstract

The epoch of Nano-biomaterials and their application in the field of medicine and dentistry has been long-lived. The application of nanotechnology is extensively used in diagnosis and treatment aspects of oral diseases. The nanomaterials and its structures are being widely involved in the production of medicines and drugs used for the treatment of oral diseases like periodontitis, oral carcinoma, etc. and helps in maintaining the longevity of oral health. Chitosan is a naturally occurring biopolymer derived from chitin which is seen commonly in arthropods. Chitosan nanoparticles are the latest in the trend of nanoparticles used in dentistry and are becoming the most wanted biopolymer for use toward therapeutic interventions. Literature search has also shown that chitosan nanoparticles have anti-tumor effects. This review highlights the various aspects of chitosan nanoparticles and their implications in dentistry.

## 1 Introduction

Over the years, scientific progress in the field of biomedicine has paved the way for the evolution of newer nano-biomaterials which have proved to be progressively efficient and biocompatible. The process of extraction of marine-based nano-biomaterials is at its peak with lot of relevant research being conducted on the same (Bonderer et al., 2008; Ghosh and Urban, 2009). Recent literature search has shown that marine based nano-biomaterials like chitosan is used extensively in medical and dental fields (Ladet et al., 2008; Jones, 2010). As stated in the “European Commission’s Recommendation”, nanomaterial can be elaborated as a natural or synthetic material incorporated with particles, in a bound or unbound state, where 50% or more of the particles are in the range of 1–100 nm (Raura et al., 2020). One of the most commonly used nanoparticle in dentistry is silver (Ag) which has been used in contrasting forms of carbon substrates and ion-oxide species. Nanoparticles are procured with unique physiological and chemical properties like nano-size, better chemical wettability and reactivity and larger surface to volume ratio for better bonding characteristics (Boverhof et al., 2015). These properties of the nanoparticles have been immensely used in treatment of oral—health related problems like treatment of dentinal hypersensitivity, eradication of oral biofilms, diagnosis and treatment of oral cancers.

**FIGURE 1:**
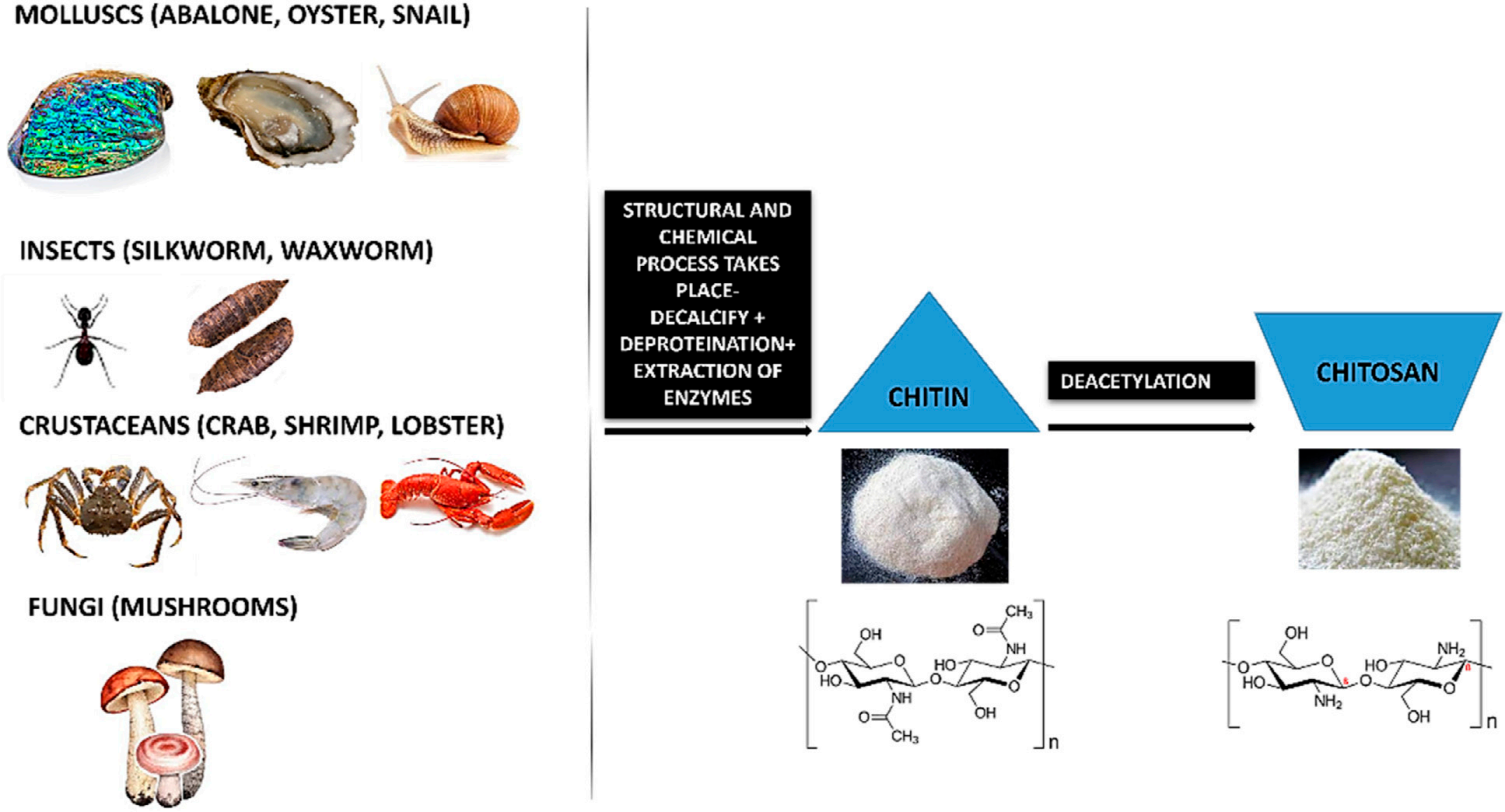
Diagrammatic illustration of de-acetylation process of chitin to chitosan.

The chemical nature and properties of chitosan like biodegradability, non-toxicity and bioacompatibility lends it to be sustainable. Chitosan is a natural nanopeptide obtained from the purification of chitin which is the main ingredient found in the exoskeletons of marine crustaceans like crabs and prawns (Paul and Sharma, 2004; Younes and Rinaudo, 2015). Other major sources of chitin include fungi (Blumenthal and Roseman, 1957; Merzendorfer, 2011), insects like silkworm, waxworm, etc (Finke, 2007; Mohan et al., 2020), certain spore-bearing plants like mushrooms, fungi (Wu et al., 2004; Vetter, 2007; Ifuku et al., 2011; Nitschke et al., 2011) and molluscs like snail, oysters, etc (Kurita, 2001; Manni et al., 2010; Rasti et al., 2017; Taser et al., 2021). This macromolecule is processed by the repeated formation of D-glucosamine, which is further extracted from de-acetylation of chitin which is a byproduct of marine shells. During the manufacturing process, the shells and the exoskeleton of these marine creatures undergo de-proteinization to form insoluble chitin (CH) which is then converted to CS (Chitosan soluble under acidic conditions) by the removal of acetyl groups (Figure 1).

**FIGURE 2:**
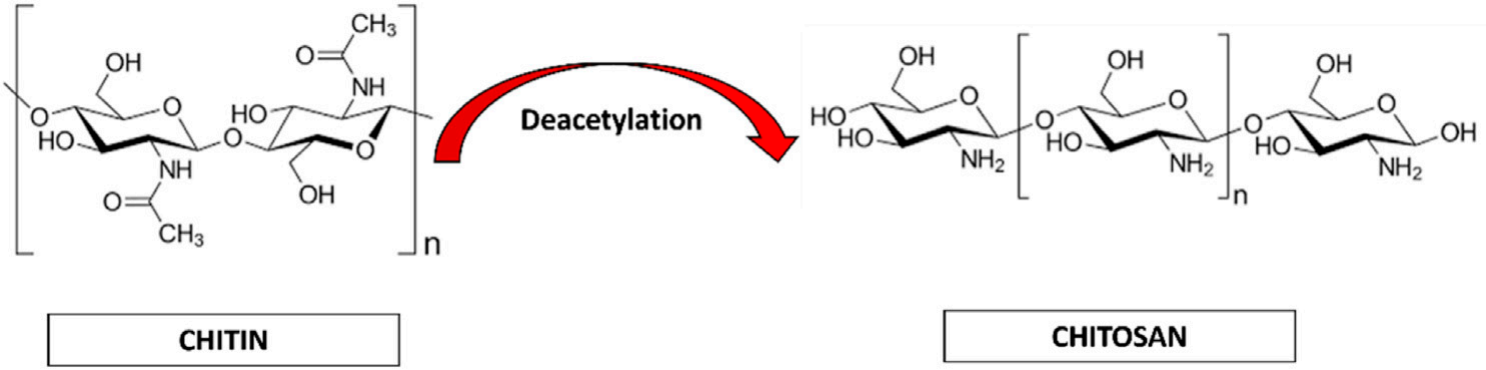
Chemical representation of Chitin to Chitosan.

Chitin (CH) is considered as the second most abundant polysaccharide after cellulose (Elieh-Ali-Komi and Hamblin, 2016). This natural amino-polysaccharide copolymer is the building block of the exoskeleton of the marine crustaceans giving them durability and stability against the natural forces. Through enzymatic de-acetylation, chitosan (CS), derivative of chitin is formed. Chitosan is a natural fiber, analogous to cellulose and cannot be digested. This biomaterial is natural, biocompatible, hydrophilic and has a broad antimicrobial and antibacterial spectrum. These natural occurring biopolymers (Chitin and Chitosan) are profusely being used for biomedical applications. Table 1 summarizes the characteristic differences between chitin and chitosan (Imai et al., 2003; Eijsink et al., 2010; Azuma et al., 2015). Chitosan (CS) is composed of N-acetyl glucosamine and glucosamine polymer units (Figure 2) which is derived from Chitin (CH).

**TABLE 1:** Chemical analysis of Chitin (CH) and Chitosan (CS).

Characteristics	Chitin (CH)	Chitosan (CS)
Molecular weight	Mw > 1000 kDa	Mw > 100 kDa
Chemical name	β-(1–4)-poly-N-acetyl-D-glucosamine	(1,4)-2-Amino-2-deoxy- beta-D-glucan
Empirical formula	(C_8_H_13_NO_5_)_n_	(C_6_H_11_NO_4_)_n_
Water solubility	Water insoluble	Poorly soluble
Sources	Exoskeleton of marine crustaceans	Derivative of Chitin
Enzymes for synthesis	Chitin synthase	Chitinase

Chitosan (CS) has wide range of commendable properties that have been used as a marker for biomedical research. Chitosan has been shown to have positive response for osteo-conductivity when amalgamated with bioactive compounds like Poly-caprolactone (Hayashi et al., 2007; Ignatova et al., 2007; Sarasam et al., 2008; Fakhri et al., 2020). These distinctive characteristics have made a remarkable entry in the field of tissue engineering and biomedical research (Guo et al., 2006; Panahi et al., 2017; Farhadian et al., 2018; Shrestha, 2018). Additionally, Chitosan has also been used as a scaffold substrate for regenerative medicine (Shi et al., 2006; Vázquez et al., 2015) and substratum for growth factor delivery for wound healing (Caetano et al., 2015; Vijayan and Kumar, 2019).

Chitosan is considered to be the only cationic poly-aminosaccharide which can be chemically altered based on the property and function (Fakhri et al., 2020). The degree of deacetylation has a strong influence on its physio-chemical-biological nature. Chitosan and it is by products like chitosan oligosaccharides are similar in nature. These nano-biopolymers can be broken down into simpler compounds through enzymatic process which are biocomapatible and based on required application, they can be modified through chemical or enzymatic reactions to variegated smaller conjugates and various forms like gels, fibers, and sponges (Xia et al., 2011; Qin and Li, 2020). Chitosan and its analogues based on their multitudinous features like cross-linking can be a vital source for the conglomerates of various biomedical materials (Pichayakorn and Boonme, 2013; Li et al., 2014).

**FIGURE 3:**
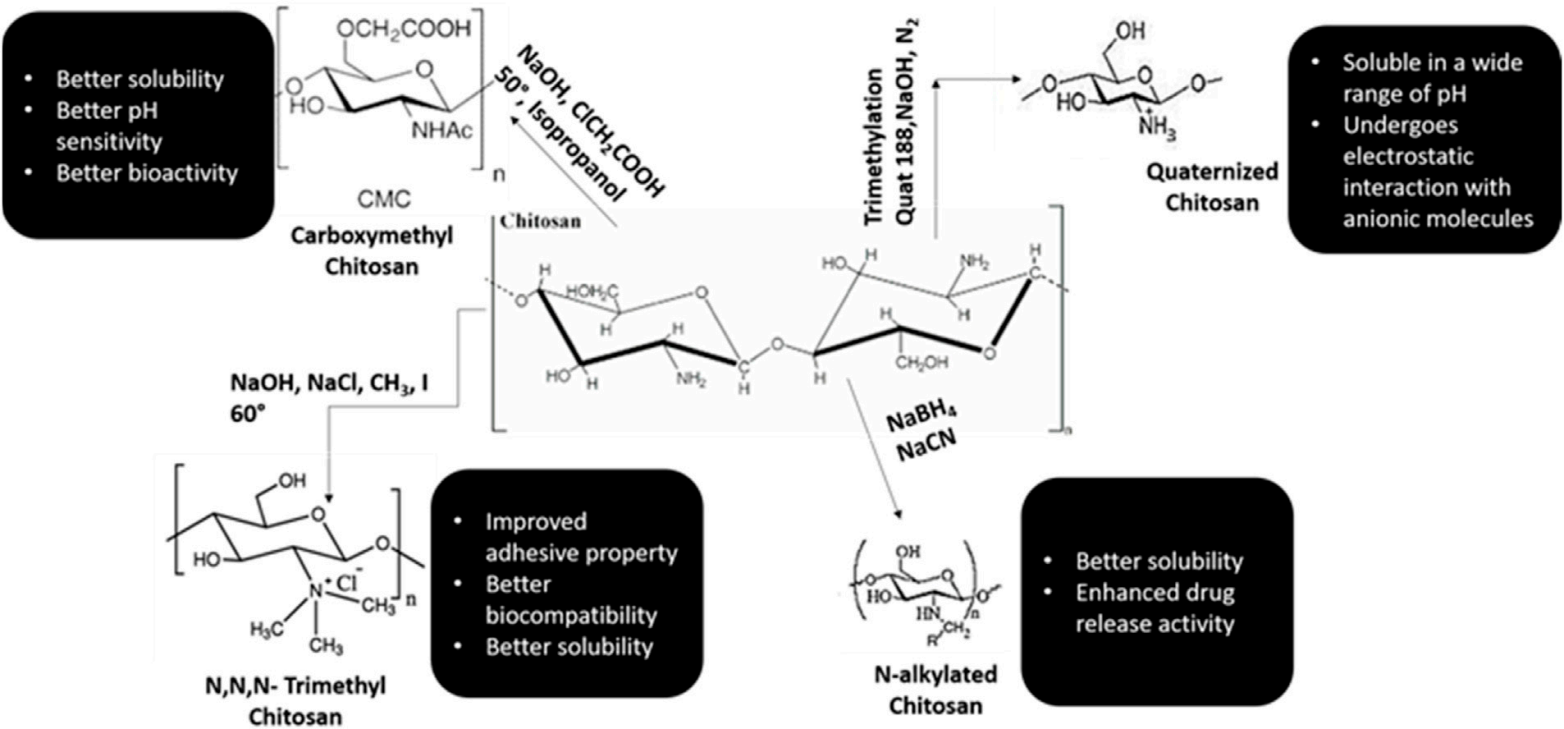
Synthetic monologues of Chitosan.

Chitosan is well known for its antimicrobial activities, but there are several theories based to this property of chitosan (Rabea et al., 2003; De Carvalho et al., 2011). One theory explains that when chitosan comes in contact with bacterial cell wall, it displaces the calcium ions of the cell membrane resulting in destruction of the membrane (Yadav and Bhise, 2004). Various literature has shown that chitosan is an effective anti-plaque agent and enhances the periodontal health by minimizing the colonies formed by *Porphyromonas gingivalis, Actinobacillus actinomycetemcomitans* and *Prevotella intermedia* (Ikinci et al., 2002; Hanes and Purvis, 2003; Akncbay et al., 2007). In animal studies, chitosan has manifested high potency of biocompatibility and shown to have positive response with implantation of nanomaterials (Levengood and Zhang, 2014; Oryan and Sahvieh, 2017; Li et al., 2021; Sivanesan et al., 2021). Various synthetic monologues of chitosan and their properties are categorized in Figure 3.

The framework of dental materials still holds a place for improvement and lot of research has been carried out for the scope of amelioration. The aim of this review is to highlight the applications of chitosan in dentistry and emphasize its importance in the treatment of various oral diseases. The bioactive properties of chitosan help in synthesis of various drugs and scaffolds for pulpo-dentinal regeneration. Chitosan has shown excellent oseteoconductivity, proliferation of osteoblasts, and mesenchymal cells thereby inducing *in vivo* neovascularization. (Kim et al., 2008; Costa-Pinto et al., 2011; Saravanan et al., 2013). Chitosan with the above-mentioned properties, makes it the most suitable component for tissue engineering. (Saranya et al., 2011; Sacco et al., 2018; Islam et al., 2020).

Chitosan is the only polycationic nanopolymer and its charge frequency hinges on the degree of acetylation and the pH condition of the media. The solubility of the chitosan depends on molecular weight and acetylation degree. High molecular weight chitosan molecules are easily soluble in acidic media. Hence enormous number of chitosan synthetic derivatives with increased solubility are produced. (Saranya et al., 2011).

**FIGURE 4:**
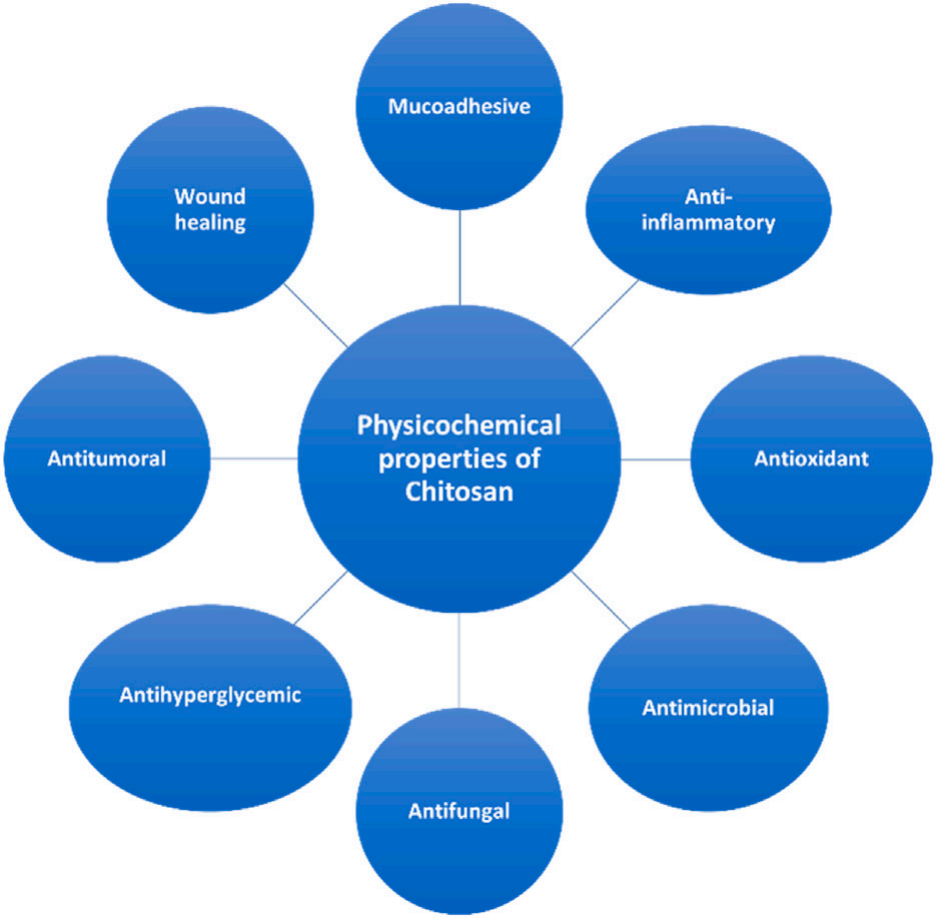
Summarization of properties of Chitosan.

The physiochemical properties of Chitosan are shortlisted in Figure 4.

## 2 Bio-dental applications of chitosan and its derivatives

Chitosan distinctive properties like biocompatibility (Zhang et al., 2002; Rodrigues et al., 2012; Norowski et al., 2015; Elieh-Ali-Komi and Hamblin, 2016), bioactive nature (Saranya et al., 2011; Prabaharan, 2014; Ainola et al., 2016; Sacco et al., 2018; Islam et al., 2020), antifungal and antimicrobial (Goy et al., 2009; Sahariah and Másson, 2017; Hassan et al., 2018; Kim, 2018; Yilmaz, 2020), anticancer activity (Wimardhani et al., 2014a; Adhikari and Yadav, 2018; Kim, 2018; Alamry et al., 2020) and ability to whisk with other materials.

Chitosan is a natural biopolymer which is easily available and extracted from natural sources. Its biomedical nature makes it one of the most efficient natural nanoparticle which can be used in dentistry. The anti-inflammatory, antifungal, antibacterial, anodyne effect, mucoadhesiveness, osseintegrative property makes it most viable material to be integrated in dentistry. It was also observed that when chitosan was laser coated with chitosan, the osteoblastic activity was enhanced which helps in better remineralization effects. Chitosan, when modulated with apatite coating, enhanced the bioactivity of chitosan which improves the physiological response. (Zhang et al., 2002; Rodrigues et al., 2012; Norowski et al., 2015).

### 2.1 Classification of nanoparticles in dentistry

**FIGURE 5:**
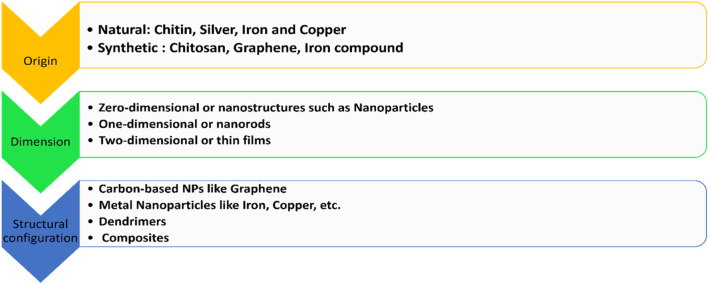
Classification of various nanoparticles used in dentistry.

Nanoparticles are classified on the basis of origin, dimension, and structural configuration (Hassan et al., 2018; Raura et al., 2020). The classification is summarized in Figure 5.

### 2.2 Synthesis of nanoparticles in dentistry

**FIGURE 6:**
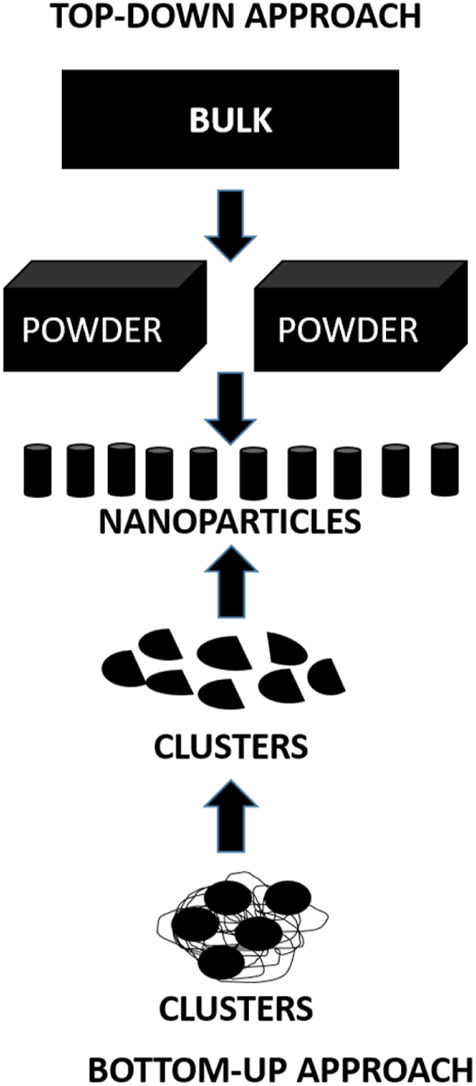
Diagrammatic representation of synthesis of nanoparticles.

There are two main approaches for the synthesis of nanoparticles (Figure 6):

1) **Top-down approach**: In this approach the bulk of the material is made to shrink to a nanoscale structure with specialized treatments like grinding, ablation, etching, and sputtering. These techniques are used for manufacturing micron sized particles. This is a simpler technique involving miniaturization of the bulk material to a small sized structure with desired properties. The basic drawback of this technique is the imperfection of the surface architecture. Nanowires made by lithography is an example of top-down approach (Yanat and Schroën, 2021; Abid et al., 2022; Indiarto et al., 2022).
2) **Bottom-up approach**: In this latter approach the material is made to undergo chemical reactions. This technique is economical, and boasts of reduced wastage of the material. This refers to building-up of the material; i.e., atom-by-atom, molecule-by-molecule, or cluster-by cluster. Some of the well-known bottom-up techniques are organo-metallic chemical route, revere-micelle route, sol-gel synthesis, colloidal precipitation, hydrothermal synthesis, template assisted sol-gel, electrodeposition etc. Luminescent nanoparticles are an example of bottom-up approach (Yanat and Schroën, 2021; Abid et al., 2022; Indiarto et al., 2022).

Besides these, there are some physical, chemical and biological methods for synthesis of nanoparticles which are enlisted in Table 2 (Yanat and Schroën, 2021; Abid et al., 2022; Indiarto et al., 2022).

**TABLE 2:** Physio-Chemico-Biological methods for synthesis of Nanoparticles.

Physical methods	Chemical methods	Biological methods
High energy Ball milling	Sol-Gel synthesis	Microorganisms assisted biogenesis
Inert Gas Condensation	Micro-emulsion Technique	Bio template assisted biogenesis
Pulse Vapor Deposition	Hydrothermal synthesis	Plant extracts assisted biogenesis
Laser Pyrolysis	Polyol synthesis	
Flash Spray Pyrolysis	Chemical Vapor synthesis	
Electro spraying	Plasma enhanced Chemical Vapor deposition	
Melt mixing		

Chitosan is cationic polymer and is efficacious against fungi and bacteria. This microbial action is attributed to the reactive hydroxyl groups at the C-3 and C-6 positions, the structure, physicochemical traits, and environmental factors of chitosan. Chitosan with it is High-MW, potential antimicrobial effects included serving as a chelator of critical metals, inhibiting nutrients from being taken up extracellularly from cells, and changing cell permeability because it is typically unable to permeate the cell wall and cell membrane. Nevertheless, low-MW chitosan affects RNA, protein synthesis, and mitochondrial function in addition to having extracellular and intracellular antibacterial activity. Moreover, the kind of bacteria that chitosan is targeting greatly influences its manner of antimicrobial action. (Ke et al., 2021).

1) Antimicrobial Activity against Bacteria

The cell wall structures of Gram-positive and Gram-negative bacteria differ significantly; Gram-positive bacteria have thicker peptidoglycans, whereas Gram-negative bacteria are more abundant in lipopolysaccharides (LPS). Because LPS is frequently linked to phosphorylated groups, Gram-negative bacteria have a greater negative charge than Gram-positive bacteria. When the pH of the surrounding environment is lower than 6.5, cationic chitosan can attach to phospholipids on more negatively charged cell surfaces. Gram-negative bacteria may be more sensitive to chitosan than Gram-positive bacteria, according to certain theories.

Gram-positive bacteria’s teichoic acids are negatively charged as well because of the phosphate groups that are present in their structure. Nevertheless, *Staphylococcus aureus* developed a greater resistance to chitosan with loss of the teichoic acid production pathway, suggesting that chitosan’s mechanism of action involves more than just electrostatic interactions. Interestingly, research have shown that DNA transcription can be inhibited by chitosan (≤50 kDa) that can penetrate the cell wall. Therefore, even though chitosan’s molecular size (MW) is crucial for targeting, chitosan’s structure—rather than its MW—determines whether it has extracellular, intracellular, or both extracellular and intracellular antibacterial action.

2) Antimicrobial Activity against Fungi

Chitosan has been demonstrated to have fungicidal effects on a variety of human and plant fungal diseases. The way chitosan interacts with the cell wall or membrane is mostly responsible for its antifungal qualities. However, the MW and level of deacetylation (DDA) of chitosan, the pH of the solvent, and the kind of fungus being targeted are all strongly correlated with the minimum inhibitory concentrations (MICs) of chitosan against fungi. Additionally, it has been suggested that there may be a positive correlation between the amount of unsaturated fatty acids on the cell membrane and chitosan susceptibility. This is because a higher amount of unsaturated fatty acids promotes improved membrane fluidity, which increases the negative charge on the membrane. The contrasting traits between chitosan-resistant and chitosan-sensitive The presence of unsaturated fatty acids in cell membranes is associated with strains of Neurospora crassa. Low-MW chitosan has the ability to pierce both the cell surface and wall, which inhibits the creation of proteins and DNA/RNA.

### 2.3 Applications of chitosan in dentistry

**FIGURE 7:**
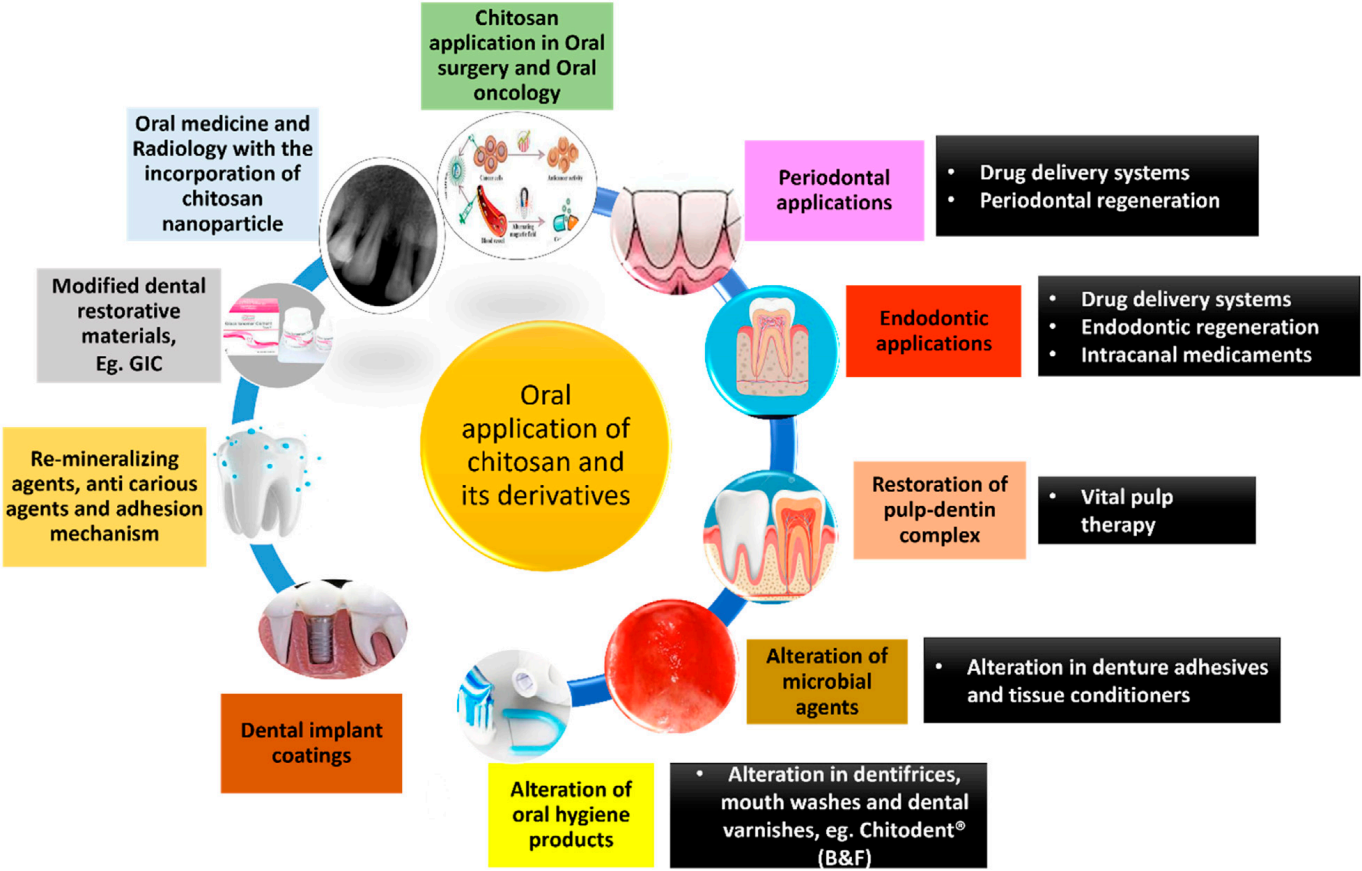
Applications of chitosan and its derivatives in dentistry.

Chitosan has a plethora of applications in the field of dentistry. A schematic diagram summarizing the same has been given below (Figure 7). (Agrawal et al., 2023; Arora et al., 2023; Nava Juárez, 2023; Paradowska-Stolarz et al., 2023; Qu et al., 2023; Minervini et al., 2024)

#### 2.3.1 Oral drug delivery systems

A lot of research has been conducted to confirm the potentiality of chitosan as an oral drug carrier. The basic aim of drug delivery analogues is to produce controlled and sustained release of drugs with prolonged contact time for a specified target with reduced dosage. This results in improving the drug efficacy and reduced side effects of systemic administration (Zivanovic et al., 2007; Priyadarsini et al., 2018). Research has shown that Chitosan-based composites (CBCs) are used to make full-bodied drug delivery carrier systems that have high mechanical strength; maintain good contact time and sustain release of the drug when they are in close contact with the oral mucosa. Chitosan based composites have been used in treatment of oral diseases (Dhand et al., 2015; Huang et al., 2016; Jeyaraj et al., 2019; Sanap et al., 2020).

The Food and Drug Administration (FDA) has approved Chitosan to be used as food supplement. Oral Chitosan nanospheres are non-toxic in nature and help in prolonged activity of drug at the site of pathology (Saboktakin et al., 2010; Stenhagen et al., 2019; Hu et al., 2020). Chitosan has been shown to have promising ingredients for drug delivery of various organic molecules like DNA, RNA, and various growth factors (Zhang et al., 2011; Soran et al., 2012; Abdel Mouez et al., 2014; Ding et al., 2017).

The proton-amino groups on D-glucosamine of the CS microstructure undergoes electrostatic linkage with negatively charged mucus layer and then invades the deepest layers of the epithelium (Kumari and Singh, 2013; Singh et al., 2013). Based on the mucoadhesive property, chitosan can be used as vehicle for drugs which are administered through various routes like nasal, buccal, ocular, and pulmonary (Keegan et al., 2012; Wang et al., 2013). The insoluble nature of chitosan can be modified through chemical alterations like carboxymethylation, acetylation, thiolation, quaternization, etc (Aguilar et al., 2019; Bakshia et al., 2019). Modified chitosan such as Quaternized chitosan with positive ions, thiolated chitosan obtained by chemical alteration of amino groups with thioglycolic acid, carboxymethyl chitosan and N-acylated chitosan are profusely used in pharmaceutical industry due to increased solubility, high pH, high mucoadhesive property and improved drug penetration (Khutoryanskiy, 2011; Bernkop-Schnürch and Dünnhaupt, 2012; Mansuri et al., 2016; Bakshia et al., 2019). CS based drug delivery systems are highly used in the treatment of dental caries, periodontitis, pulp space therapies and prolonged anesthesia.

The adverse effects of systemic administration of drugs have resulted in poor patient compliance; so, to overcome this, local drug deliveries for periodontal pockets has come to the forefront. Local drug delivery systems permit prolonged release of the drug in periodontal pockets with a long contact time resulting in better treatment outcome (Chen et al., 2017; Zhang et al., 2018a; Wang et al., 2020). These systems act as an adjunct to oral prophylaxis and decrease the systemic adverse effects.

Chitosan nanoparticles have been shown to have anti-inflammatory effects on human gingival fibroblasts by reducing the number of receptors on inflammatory cytokines and chemokines such as IL-1β, TNF-αand CXCL-8. Various studies have shown that human gingival fibroblasts tend to have increased metabolic ability and cellular viability in the presence of chitosan nanoparticles which help in reconstitution of gingival tissue (Goodson et al., 1985; Mahmood et al., 2017; Shen et al., 2017).

Chitosan microspheres are spherical patches ranging from few micrometers to 1000 µm and contain medicinal agents in a polymerized matrix. These patches protect against salivary digestive enzymes and can be applied in mucosal membranes and sub-gingival sites (Greenstein and Polson, 1998; Joshi et al., 2016). Recently, there are micro-formulations of chitosan with anionic bio-particles like alginate, xanthan gum, hyaluronic acid and pectins. These micro-formulations are termed as polyelectrolyte complexes (PEC) which provide sustained release of drugs and are less toxic when compared to cross-linked polymers (Agnihotri et al., 2004; Sinha et al., 2004; Arancibia et al., 2013; Silva et al., 2013; Babrawala et al., 2016). Yadav and co—workers in a study used chitosan, calcium and sodium alginate combination microspheres to contain antibiotics like ornidazole and doxycycline and proved the efficacy, muco-adhesive activity and biodegradability of these microspheres (Yadav et al., 2018). Many attempts to combine microspheres and hydrogel to provide two-layered barrier system has been tried (Zhao et al., 2014).

Chitosan nanoparticles have propitious results when treating oral pathologies. These group drug delivery devices benefit from the small particulate size and the innate characteristics of the polymer. Owing to sizing of the nanoparticles, these particles can penetrate through impervious barriers and also contact the tissues over a larger surface area (Fakhri et al., 2020). These chitosan nanoparticles protect the gastrointestinal tract from enzymatic degradation and acidic environment (Wang et al., 2011; Alcaraz et al., 2016). The beneficial aspects of chitosan nanoparticles encapsulated with antibiotics like doxycycline (Madi et al., 2018; Hu et al., 2019), silver nanocrystals (Xue et al., 2019), tetracycline (Parsa et al., 2019) and ciprofloxacin (Zhao et al., 2013) have been studied and confirmed. A lot of research is conducted on chitosan/PLGA (Polylactic co-glycolic acid) nanoparticles and has shown to have improved stability, enhanced drug release and non-toxic behavior (Lu et al., 2019). Chitosan laden PLGA, Lovastatin and Tetracycline nanoparticles have been explicated and have been revealed to induce osseous formation, alkaline phosphatase activity, biocompatibility, antibacterial activity and controlled and sustained release of these drugs to treat existing pathological condition (Lee et al., 2016).

Chitosan nanofilms are gaining popularity and are effectively used in the interproximal pockets (Salari et al., 2018). The advantages of films as drug delivery devices include biodegradability and ease of placement without intervening with daily activities. The films can be altered and adjusted according to the size of the defect site (Soskolne, 1997). The placement of films in the oral cavity gets disturbed due to saliva and its lubricating efficacy. Muco-adhesiveness is considered to be a pre-requisite property for the manufacturing of nanofilms. In a study by Ghafar et al., thiolated chitosan based nanofilms were introduced for the release of calcium fluoride for dental caries and for the release of lignocaine for diminishing pain. Based on this study, it was found that the thiol groups from the films get released and interact with the oral mucosa for long duration increasing the contact time of the drug, and also regulating the release of fluoride. Hence, thiolated chitosan based nanofilms can be suggested for oral problems (Ghafar et al., 2020). Chitosan–alginate complex films are durable, have improved physical and mucoadhesive properties. These films are said to have higher alginate content with increased concentration which aids in slow drug release (Kilicarslan et al., 2018). CS-alginate films have been used to incorporate natamycin and silver nanoparticles (da Silva et al., 2013). Chitosan laden with risedronate and zinc hydroxyapatite (CRZHF) films are introduced for treatment of periodontitis as anti-resorptive medicament. These CRZHF films are flexible and have good mucoadhesive strength resulting in hard detachment after the placement of the film into the periodontal pockets. Clinical trials with CRZHF have shown increased alkaline phosphatase activity, resulting in bone formation and improved successful treatment outcome (Khajuria et al., 2018). Chitosan-based films with local anesthetics have been implemented to relieve pain and discomfort in patients. Chitosan/collagen films incorporated with lidocaine, tetracaine and benzocaine for effective delivery of local anesthetics have also been tried. These films have good mucoadhesion, and improved flexibility (DiMartino et al., 2019).

Another form of drug delivery systems are chitosan gels. These gels include drug macromolecules incorporated into the polymeric structure and help in controlled and sustained release of the drug. These hydrogels are manufactured by chemical crosslinking to form permanent bonds and physical crosslinking to form provisional bonds. These cross-linked hydrogels have good viscosity, high mucoadhesive properties, better injectability and prolonged release of the drug at the site (Bhattarai et al., 2010). Chitosan gels combined with 15% metronidazole have been used as an adjunct to mechanical debridement for periodontitis to improve the treatment outcome (Akıncıbay et al., 2007). Recently, thermosensitive gels have been introduced into dentistry; these hydrogels reform their gelation nature according to alterations in temperature and display sol-to gel transformation when administered into the body. The first thermosensitive hydrogel composed of chitosan, quarternized chitosan and β-glycerophosphate was introduced against periodontal pathogens like *P. gingivalis* and *P. intermedia* (Akncbay et al., 2007; Ji et al., 2010). Chitosan, β-GP hydrogel in injectable form packed with ornidazole and BMP-7 was used for periodontal regeneration of furcation defects (Bansal et al., 2018). Bacterial plaque causes low pH environment in the oral cavity which can alter the release of the drugs from the hydrogel *in situ*. To overcome this, pH sensitive hydrogels like N-carboxymethyl chitosan-based hydrogel, injectable chitosan-grafted-dihydrocaffeic acid/oxidized pullulan hydrogel, etc. have been introduced. These hydrogels are pH- dependent and exhibit good swelling behavior, drug release and muco-adhesiveness. These hydrogels are well-known for cancer therapy and tumors which create an alteration in pH in the oral environment (Liang et al., 2019). Hydrogels which control the drug release have been found to be very efficient with characteristics like eletro-responsiveness and pH sensitivity and are manufactured by implanting polyaniline onto chitosan and crosslinking with oxidized dextran (CS-P/DO). These hydrogels are cyto-compatible and biodegradable (Alinejad et al., 2016).

Chitosan fibers also form one of the recently introduced drug delivery systems. Chitosan has known to have high fiber-forming property. Chitosan fibers are constructed through electrospinning process producing fibers ranging from nanometers to micrometers. CS fibers are extravagantly used for neural and osseous tissue engineering. These fibers tend to have low molecular weight; hence they are reticulated with epichlorohydrin, hydroxyapatite, PLGA, poly-caprolactone, cellulose, polyvinyl alcohol, etc. (Wei et al., 1992; Tanir et al., 2014). The Electrospun chitosan fibers with drug are potentially used in guided tissue engineering. Chitosan-Polycaprolactone cross-linked with metformin membrane stimulates bone formation, alkaline phospahatase activity and mineralization of bone mesenchymal stem cells (Zhu et al., 2020). Chitosan- Gelatin nano-carriers laden with calcium hydroxide are used for endodontic infections for sustained release of calcium hydroxide for longer duration. The combination of chitosan with calcium hydroxide has shown excellent antibacterial activity against endodontic pathogens like *Enterococcus faecalis* (Shaik et al., 2014; Malinowska et al., 2017). Table 3 mentions a list of a few of the many studies on nanoparticle drug delivery modes with antibiotics.

**TABLE 3:** Studies illustrating on the drug delivery modes with antibiotics.

Ref	Type of delivery system	Biomaterial/Polymer	Drug inoculated	Conclusions
Goodson et al. (1985)	Hydrogel	Chitosan	5% Tetracycline	• Great antibacterial activity against Gram-negative and Gram-positive bacteria
				• Can be used as wound dressing
Greenstein and Polson (1998)	Nanospheres	Chitosan	Ciprofloxacin	• Excellent antibacterial activity against *E. coli*
Agnihotri et al. (2004)	Polyelectrolyte films	Chitosan + Alginate	Clindamycin	• Good drug delivery system for periodontal therapy
Ghafar et al. (2020)	Nanoparticle	Chitosan + Dextran	IL-17RB siRNA and doxorubicin (DOX)	• Co-delivery of IL17RB siRNA and DOX have shown excellent results in the treatment of breast cancer
Khattak et al. (2019)	Intracanal medicament	Chitosan	Calcium hydroxide + Triple antibiotic paste	• Highly effective against *E. faecalis* and C. albicans to treat endodontic infections

#### 2.3.2 Alteration of antimicrobial agents

The positively charged amino groups of N-acetyl glucosamine combines with negatively charged ions of the bacterial cell wall containing lipids, phospholipids, carbohydrates and proteins (Kong et al., 2010). This basis remains the same for fungal and viral micro-organisms. The underlying mechanism for the antibacterial action of chitosan is still not clear, but the theories state that the amino groups interact with the negatively charged particles of bacterial cell wall leading to cell wall leakage, increasing the permeability and ultimately leading to cell destruction (Matica et al., 2019). Another theory for the mechanism of action of chitosan believed, is that the low molecular weight of chitosan can penetrate the bacterial cells and impede the bacterial activities like RNA and protein synthesis. High molecular weight molecules of chitosan particles >100 kDa, tend to deposit a polymer material around the cell membrane and cuts down the nutrient supply (Younes et al., 2014). However, the bio-properties of chitosan are magnified by decreasing the deacetylation process and modifying the pH of the environment (Lim and Hudson, 2004; Li et al., 2016).

On the whole, intensifying the positive charges of chitosan molecules increases the electrostatic reciprocity with the cellular contents and thereby increases the antimicrobial efficacy of chitosan. Certain cross-linking methods like carboxymethylation, sulfonation, quaternization, and phosphorylation enhance the solubility of chitosan and increase its antimicrobial efficiency (Lim and Hudson, 2004; Xu et al., 2011). Quaternized chitosan and its derivatives have been extensively studied on the aspects of their antimicrobial efficacy and shown to have the best results (Zhang et al., 2002; Ignatova et al., 2007; De Carvalho et al., 2011; Wang et al., 2016; Xu et al., 2018). Ammonium salts of Chitosan are shown to be highly effective against *E. coli* and *S. aureus* (Kim et al., 1997).

Chitosan and its compounds are effective on fungal and bacterial substrates. Congregation of literature has divulged the efficacy of chitosan film on medical instruments against contamination (Ghosh et al., 2011; Islam et al., 2019). Based on the efficacy, chitosan can be cross-linked to tissue conditioners or denture adhesives and help in the prevention of denture stomatitis (Lee et al., 2018; Namangkalakul et al., 2020). Tissue conditioners made of chitosan and chitosan oligosaccharide are excellent alternatives for treatment of denture stomatitis. High anti-microbial property and water solubility of Chitosan oligosaccharide makes it an ideal choice for reducing *Candida albicans* infections. Tissue conditioners made of quaternized chitosan can be used as provisional lining materials for treating denture stomatitis (Saeed et al., 2019).

Chitosan has played a vital role in the treatment of endodontic infections. Calcium hydroxide cross-linked with chitosan as intracanal medicament has been effective in reducing the periapical infections. Chitosan has anti-biofilm property which aids in controlling the microbial load and effective against *E. faecalis, Streptococcus mutans*, and various other microbes (Elshinawy et al., 2018; del Carpio-Perochena et al., 2017; Loyola-Rodríguez et al., 2019; Ganss et al., 2011; Young et al., 1997; Ganss et al., 2014). Antibacterial efficacy of endodontic sealers incorporated with chitosan has been seen to be effective for longer periods of time (Pini et al., 2020).

#### 2.3.3 Alteration of oral health products

Use of oral hygiene products like toothpastes, brushes and mouth wash plays a very key role in oral hygiene maintenance. Dentifrices are known to fend off the demineralization effects of tooth substrate due to acidic drinks. Numerous dentifrice formulations have been studied and enlisted in table 4. Various toothpaste preparations containing nanoparticles with hydroxyapatite, 5% KNO_3_, etc., aim at providing fluoride (F) release towards tooth re-mineralization of enamel substrate. In a study by Ganss et al., a non-fluoride, chitosan-based dentifrice (Chitodent^®^ (B&F)) was investigated and showed significant reduction in tooth tissue loss (Costa et al., 2014). This was attributed to the cationic character of chitosan combined with low pH, and affinity to bind to negatively charged structures like enamel and dental biofilm. The presence of nanoparticles like chitosan helps in formation of organic protective layer over the mineralized structures (Costa et al., 2014; Farias et al., 2019; Pini et al., 2020). Certain toothpastes containing Strontium (Sr) and potassium nitrate have shown to diminish the erosion of dentin (Covarrubias et al., 2018). F/Sr containing toothpastes combined with chitosan tend to have anti-erosive and anti-abrasive properties. Pini et al., in their study concluded that increasing the viscosity of chitosan to F/Sr toothpaste, helps in complete inhibition of enamel tissue loss thereby retaining the enamel surface (Costa et al., 2014).

**TABLE 4:** Studies highlighting the effectiveness of chitosan modified oral hygiene aids.

Ref	Type of study	Type of oral hygiene aid	Chitosan-coupled groups	Comparative groups	Conclusions
Ganss et al. (2011)	*In-vitro*	Tooth paste	Fluoride-free CS-toothpaste (Chitodent)	NaF-toothpastes, NaF/KNO_3_, NaF/HA, Zn/carbonate/HA, SnF_2_/NaF, SnCl_2_/NaF,SnF_2_	SnF_2_ most effective
					Chitodent reduced tissue loss by 30%
Ganss et al. (2014)	*In-vitro*	Tooth paste	AmF/NaF/SnCl_2_/CS 0.5%	NaF, SnF_2_ gel, toothpaste	AmF/NaF/SnCl_2_/CS showed significant reduction in tissue loss with or without brushing
Pini et al. (2020)	*In-vitro*	Tooth paste	F/Sn/CS-toothpaste	F/Sn-toothpaste	Chitosan with higher viscosity of (1000 mPas) showed the best anti-erosion/abrasion effects
Costa et al. (2014)	In- vivo	Mouthwash	HMW CS 0.4 v/v	Chlorhexidine	Chitosan incorporated mouthwash was highly effective against Streptococci and Enterococci with the lowest cytotoxicity
			LMW CS 0.4 v/v		
Farias et al. (2019)	*In-vitro*	Mouthwash	MPEO/CS/biosurfactant	Biosurfactant/MPEO, fluoride-free mouthwash	Mouthwash containing chitosan showed high antimicrobial activity against cariogenic bacteria with least toxicity
			Biosurfactant/CS		
Covarrubias et al. (2018)	*In-vitro*	Varnish	CSnP/NaF, CSnP	Miswak, Miswak/F, Propolis, Propolis/F, NaF	NaF coupled with chitosan nanoparticles exhibited highest anti-bacterial and anti-demineralization activity

Recently, a lot of research has been involved in the manufacture of chitosan-based oral hygiene maintenance products like mouthwash, varnishes, nanogels (Table 4). Costa and his co-workers, studied the efficacy of chitosan based mouthwashes on biofilm formation and microbial attachment of *E. faecalis, C. albicans, S. mutans and P. intermedia* and concluded that these mouth washes are effective in controlling dental caries, periodontal problems and fungal infections. Various formulations of mouthwashes like *Mentha piperita* essential oil (MPEO) with chitosan have shown to have prolonged anti-caries effects. Currently, a new lineage of anti-carious products containing combinations of metallic compounds like silver or copper with chitosan have been experimented and documented that chitosan undergoes electrostatic interaction with tooth structure and bacterial cell wall, thereby escalating anti-biofilm property (Arnaud et al., 2010; Costa et al., 2014; Zafar and Ahmed, 2014; Wassel and Khattab, 2017; Covarrubias et al., 2018; Farias et al., 2019).

#### 2.3.4 Re-mineralization of enamel

Restoration of lost enamel is one of the most challenging tasks in dentistry since enamel is formed only once in the human body and is avascular (Zhang et al., 2018b). Various bioactive dental materials have been developed for regeneration of enamel but till date majority of them are disbelieving. Chitosan structure gets protonated with free amino groups and creates a positive charge around it. This property helps chitosan to bind to negatively charged structures like tooth enamel. In addition, chitosan can invade into the deeper layers of enamel producing mineral content and thereby helping in re-mineralization of carious lesions. Hence, chitosan-based materials help in re-mineralization of lost enamel and prevent the progression of carious lesions (Ren et al., 2019; Zhang et al., 2019). In a study, it was demonstrated that Chitin-bioglass complex helps in deposition of mineral content and refines the eroded or carious enamel surface; improves the microhardness of surface and sub-surface areas. Chitosan nanoparticles coupled with amelogenin in the form of hydrogel help calcium and phosphate ions to reorganize and form enamel like structure. (Ren et al., 2019). The above-mentioned hydrogel has got excellent anti-bacterial and re-mineralization capacities.

#### 2.3.5 Bonding to tooth structure and adhesion mechanism with chitosan

The resin-dentin bond and the longevity of the bond strength has received a lot of attention and is being researched upon. Dental materials like composites are used for bulk filling and dentin replacement which tend to cause polymerization shrinkage and involves technique sensitive steps like acid etching and bonding, and removal of smear layer (Murray et al., 2003). When there is incomplete removal of smear layer, the resin monomer does not flow in properly resulting in polymerization shrinkage and ultimately microleakage (Chen et al., 2003). To overcome this demerit, chitosan hydrogels and polymeric bio adhesives have come into demand. In a study, chitosan hydrogels incorporated with propolis, β carotene and nystatin were evaluated and shown to have increased shear bond strengths over a period of time. The shear bond strengths with respect to chitosan hydrogel have been deemed higher than conventional dentin-bonding systems (Perchyonok et al., 2013).

#### 2.3.6 Chitosan coated dental implants

The extent of osseo-integration of dental implants with alveolar bone marks the clinical outcome of the dental implants (Javed et al., 2014; Alghamdi, 2018). To improve the osseo-integration effects, numerous methods such as chemical surface treatments, and surface coating for implants have been tried and shown favorable results (Najeeb et al., 2016; Guglielmotti et al., 2019). Various bioactive layering on the surface of implants have resulted in improved bone health and osseo-integration in immunocompromised patients (Abtahi et al., 2012; Pang and Huang, 2012; Diz et al., 2013; Javed et al., 2014; Najeeb et al., 2017; Duarte et al., 2021). Multitude of studies have highlighted the beneficial aspects of chitosan coatings for dental implants (Redepenning et al., 2003; El Nady et al., 2017; Li B. et al., 2019; Alnufaiy et al., 2020; Yilmaz, 2020). The chitosan coating on dental implants tends to decrease the surface roughness, hydrophilicity which helps in apaptite formation, cell adhesion and proliferation thus increasing the bioactivity. Chitosan bio-coatings show excellent biocompatibility, no cytotoxicity and better antibacterial activity. It is also stated that increased thickness of chitosan coating on dental implants prolonged the antibacterial activity (Norowski et al., 2011). Literature has also reported that chitosan layering on dental implants reduce stress concentrations by changing the elastic modulus of the bone-implant interface (Levengood and Zhang, 2014). In addition, research has shown that antibiotics can be incorporated on the chitosan coatings for better healing around the implant area (D’Almeida et al., 2017; Cohenca et al., 2013). Antibacterial coatings on implants and medical devices have shown to have good clinical success, but still further investigation is required.

#### 2.3.7 Chitosan modified dental restorative materials

Over the recent years, a lot of noteworthy research has been tried and tested to create opportunities for bioactive dental materials. The level of destruction to the pulpo-dentinal complex dictates the treatment prognosis with the use of biomimetic materials (Drummond, 2008). However, certain flaws have been delineated which include poor adhesion, less mechanical strength when compared to ceramic or resin-based materials (Wimardhani et al., 2014a). Owing to these disparities, poor interfacial bond is created between the tooth and the material resulting in microleakage (Bentley and Drake, 1986; Ho et al., 1999).

Glass ionomers (GI) are tooth colored restorative materials comprising calcium fluor aluminosilicate glass powder and polyacrylic acid liquid. GIs bond chemically to the tooth structure and exhibits anticariogenic property with the release of fluoride (Forsten, 1994). There are numerous applications of Glass ionomers like luting/cementation of crowns and bridges, restorative materials for class V lesions and non-carious lesions like erosions, sandwich technique for class II restorations, core build-ups, atraumatic restorative treatment, lining material under composite restorations and pit and fissure sealants (Sidhu and Nicholson, 2016). Despite all the advantages, GIs have certain limitations like poor mechanical strength, low fracture toughness, low abrasion resistance and poor esthetics. Hence the application of conventional glass ionomers on high stress bearing areas is avoided (Khoroushi and Keshani, 2013). To overcome these discrepancies, experimentation has been done to integrate bioactive polymers into restorative materials and modify their properties.

Currently, nanoparticles like chitosan have been coupled with glass ionomers to modify their mechanical and antibacterial properties. The anionic groups of polyacrylic acid of Glass ionomers co-agglomerate with cationic amine groups of chitosan to form interpenetrating polymerized network. It can be predicted that inclusion of nano-chitosan particles to Glass ionomer materials would enhance the mechanical strength and anti-cariogenic property for high stress bearing area applications (El-Negoly et al., 2014; Senthil et al., 2017). It has also been proved that application of chitosan to liquid component of GIs improves the chemical adhesion and antibacterial activity (Soygun et al., 2021). Chitosan modified GIs have been clinically accepted as a root coverage material for gingival recession which promises minimal to no-genotoxicity and cytotoxicity effects and enhances the proliferation of human gingival fibroblasts (Zhou et al., 2019). It is also noted that chitosan modified GIs helps in sustained release of proteins, growth factors and bioactive polymers which is apt for vital pulp and regenerative therapies. In a study, it was reported that ion release from Chitosan modified Glass ionomers was advantageous to the tooth substrate (Mulder and Anderson-Small, 2019), and also helps in sustained release of proteins without any cytotoxic effects to pulp cells (Limapornvanich et al., 2009). Various bio-molecules like Tumor Necrosis factor, growth factors, peptides, TGFβ-1 can be applied to chitosan modified GICs to stimulate pulp regeneration (Rakkiettiwong et al., 2011).

Enormous amount of research is being carried out to modify the physical properties of various dental materials. Recently, chitosan-based nanocomposites have been developed and are claimed to have superior features like strength, heat stability, electrical conductivity, photoluminescence, antimicrobial, and biomedical features which increase the longevity of dental restorations (Diolosà et al., 2014; Kausar, 2021). In addition, chitosan modified zinc oxide eugenol cements, root canal sealers have also been investigated (Dragland et al., 2019).

#### 2.3.8 Chitosan and pulpal regeneration

Regeneration of dental pulp is one of the most unpredictable situation in dentistry. Tissue engineering approaches like stem-cell based transplantation have been experimented to simulate the dentin-pulp complex. Stem cells are harvested from various sources like dental pulp stem cells (DPSCs), stem cells from human exfoliated deciduous teeth (SHEDs) and stem cells from apical papilla (SCAP) (Huang et al., 2008). These cells have highest potential of differentiating into odontoblast like cells, high proliferation capability, and multi-cell differentiation capacity. DPSCs are easily extracted from human permanent or deciduous teeth. Transplanting human DPSCs onto the chitosan scaffold for pulpo-dentinal complex regeneration is still under research. However, cell homing via DPSCs seem to have promising results in regenerative endodontics. Some of the clinical trials have shown to form connective tissue similar to dental pulp with neovascularization and in some cases, dentin deposition was also noticed (Eramo et al., 2018).

The stimulation of mesenchymal stem cells to form odontoblastic cells forms the basis for endodontic regeneration. It has been reported that porous chitosan scaffolds when coupled with bioactive molecules like growth factors and peptides, help in release of odontoblastic markers like dentin sialo-phosphoprotein, alkaline phosphate, and dentin matrix acidic phosphoprotein which form an extracellular polymerized matrix which acts as niche for the multiplication and proliferation of DPSCs into odontoblastic cells resulting in mineralization process (Soares et al., 2018; Bakopoulou et al., 2019; Raddall et al., 2019; Moreira et al., 2021). In addition, Chitosan scaffolds enriched with signaling molecules like BMP 1and7, vascular endothelial growth factor (VEGF), brain-derived neurotrophic factor (BNF), fibroblast growth factor (FGF) and drugs like simvastatin and metformin help in inducing reparative dentin formation by promoting cell adhesion and proliferation of DPSCs (Guo et al., 2006; Jingwen et al., 2016; Schmalz et al., 2017; Retana-Lobo, 2018; Soares et al., 2018; Yang et al., 2020). Biomolecules like TGF β-1 when loaded with chitosan are proving to be excellent alternatives to calcium hydroxide as a pulp capping material since it regulates the differentiation of odontoblastic stem cells, alkaline phosphatase, OCN gene/protein expression and bio-mineralization (Farea et al., 2014; Abbass et al., 2020). SCAP/carboxymethyl chitosan/TGF-β1 scaffold is also reported to be favorable for pulp regeneration as it releases CS nanoparticles, dentin matrix protein-1 and dentin sialo-phosphoprotein (Bellamy et al., 2016).

Zhu and his co-workers brought a breakthrough in the field of vital pulp therapy. They introduced injectable Ag-BG/chitosan thermosensitive chitosan hydrogels which promote odontogenic regeneration due to their viscosity, flexibility and thermosensitive nature (Zhu et al., 2019). These hydrogels possess high antibacterial and anti-inflammatory potential and the chitosan release odontogenic markers like PGE2, TNFα, IL-1β, −6 and −8 which are ideal for endodontic regeneration (Silva et al., 2013; Aguilar et al., 2019; Zhu et al., 2019). Another innovation reported with the development of pulp regeneration was chitosan-cellularized fibrin hydrogel which led to proliferation of collagen type I and dental pulp mesenchymal cells producing 3-D collagenous network analogous to innate pulpal tissue (Ducret et al., 2019). Qin et al. in their study inoculated chitosan–metformin with Calcium phosphate cements to enhance the strength, mineralization and osteogenic potential (high alkaline phosphatatse activity and increased proliferation of DPSCs) of biomembranes (Qin et al., 2018). Chitosan along with hDPSCs have shown to have promising results for endodontic regeneration.

#### 2.3.9 Chitosan modified wound dressings

These dressings are highly effectual for controlling hemorrhage and infection after surgical operative procedures. As stated earlier, the positively charged amino groups of chitosan electrostatically interact with negatively charged elements of blood, i.e., RBCs to bring about the hemostatic property (Caetano et al., 2015; Minervini et al., 2024).

Quaternized chitosan-G-polyaniline and benzaldehyde group functionalized poly (ethylene glycol)-co-poly (glycerol sebacate) (PEGS-FA) hydrogels have high antibacterial, antioxidant, antimicrobial property, good self-healing capacity which makes it ideal for the manufacture of wound dressings. These self-healing hydrogels are bestowed with free radical scavenging capacity, adhesiveness, biocompatibility and excellent *in vivo* blood clotting capacity which stimulate the wound healing mechanism, granulation tissue formation and collagen deposition by upregulating the growth factors like Transforming Growth Factor-β (TGF- β), Epidermal growth factor (EGF) and Vascular endothelial growth factor (VEGF) (Zhao et al., 2017). It is also proved that Chitosan dressings when refined with polyphosphate and silver, escalates the hemostatic and antimicrobial activity. Chitosan-polyphosphate formulation (ChiPP) enhances blood clotting, platelet adhesion, faster thrombin generation and better absorption of blood (Dai et al., 2011).

pH-responsive hydrogel wound dressings containing quaternized chitosan/benzaldehyde-terminated pluronic F127 (PF127) coupled with curcumin have shown enhanced antimicrobial, anti-inflammatory, and improved wound healing activity. These hydrogels enhance the wound healing process by minimizing the inflammatory markers and upregulating the wound healing-related growth factors (Qu et al., 2018). Recently, a major breakthrough, i.e., in the treatment of infected wounds, a photothermal self-healing nanocomposite hydrogels with antibiotics have been introduced. These nanocomposite hydrogels are presented with N-carboxyethyl chitosan/PF127/carbon nanotube and exhibit stable hemostatic, mechanical properties, remarkable photothermal antibacterial property and an increased pH-responsive moxifloxacin release capacity to enhance the healing process (He et al., 2020).

#### 2.3.10 Chitosan based tissue engineering aspects

Periodontitis is a chronic inflammatory disease affecting the periodontium and the tooth supporting structures resulting in loss of alveolar bone and mobility of teeth. To overcome this situation, surgical approaches like guided tissue regeneration (GTR) and guided bone regeneration (GBR) can be considered. In these procedures, a scaffold barrier is placed to allow osteloblastic proliferation and osseous formation around the bone defect. The barrier obstructs the epithelial cell migration resulting in formation of long junctional epithelium *in lieu* of bone formation (Newman et al., 2011).

The basic aim of tissue engineering is to construct a 3-D scaffold which bears a close similitude to the structure of bone. Tissue engineering aims to create a regenerated tissue on polymerized decomposable scaffold as a layering for stem cell attachment, adhesion, and proliferation. The manufacture of bio-membrane should be biocompatible, non-toxic, biodegradable and should resemble the extracellular matrix (ECM) containing glycosaminoglycans, glycolipids and glycolipids for neo-regeneration of the tissue (Baranwal et al., 2018).

Natural and synthetic biopolymers are present to provide scaffolding. Chitosan and various subordinates meet the fundamental requirements of tissue engineering. It has been advocated that chitosan implantation does not provoke any immune response and the breakdown of chitosan by lysozyme upon the formation of new tissue does not produce any toxic effects. In dentistry, chitosan scaffolds are extensively used for pulpo-periodontal-bone regeneration (Li et al., 2014; Vázquez et al., 2015; Sultankulov et al., 2019). Chitosan is amenable to the characteristics of scaffold; however it lacks superior mechanical property and bioactivity which are the essential basis for osseous tissue engineering (Azevedo et al., 2014). To achieve this point, chitosan is coupled with synthetic biopolymers, growth factors and proteins to enhance the osseous regeneration with improved mechanical strength. Chitosan membrane laden with bioactive nanoparticles like hydroxyapaptite (HA), silica and tricalcium phosphate (TCP) stimulates bone formation and improves mechanical properties (Faqhiri et al., 2019). Additionally, nano-ceramic particles like Bioactive glass can be applied to chitosan to promote osteogenesis in load bearing areas (Denuziere et al., 1998). Chitosan/chondroitin sulfate/Bioactive glass nanoparticles facilitate osteogenesis *in-vivo* (Nie et al., 2019). Nanocomposite scaffolds containing chitosan-gelatin- nanobioactive glass are proven to create a sterile environment for cell attachment to promote protein adsorption and mineral deposition to promote osseous formation (Januariyasa et al., 2020). Combination of nanomaterials with bioactive growth factors on a 3-D scaffold directs the mesenchymal stem cells to differentiate to osseous formation (Guo et al., 2006; Oryan and Sahvieh, 2017).

Various studies have reported that chitosan-hydroxyapaptite combination coated scaffolds provide excellent environment for cell differentiation and proliferation promoting osseous formation similar to the original bone structure. The incorporation of nanohydroxyapatite in the scaffold increases the apatite content in the defective area which provides the basis for bone tissue engineering which led to the development of chitosan double faced membranes (Gümüşderelioğlu et al., 2020). These membranes were used for periodontal regeneration. The porous side of the membrane (in contact with bone) was loaded with nanohydroxyapatite and BMP-6, and the opposite side was laden with poly-caprolactone nanofibers to mitigate the epithelial cell migration. The porous surface induced multiplication of MC3T3-E1 preosteoblasts, while the other side of the membrane acted as a guard against epithelial cell migration (Marrazzo et al., 2016; Suneetha et al., 2020).

Injectable hydrogels have always been reliable and used as a part of tissue engineering process. Chitosan membranes in the form of injectable hydrogels do not require surgical intervention. These hydrogels are highly used for the treatment of periodontal pockets. It has been observed that PEC hydrogels coupled with chitosan and sodium alginate in a polymerized network have improved mechanical properties and biocompatibility which are requisites for osseous tissue engineering. These hydrogels tend to form porous ladder like interconnected mesh with fibrous structure on which osteoblasts proliferation is enhanced (Tatullo et al., 2017). Drug–based chitosan scaffolds have gained lot of popularity and are extensively used for bone and periodontal healing. (Agrawal et al., 2023; Arora et al., 2023; Nava Juárez, 2023).

Cell-based approaches are becoming a trend in regenerative medicine to treat health related conditions. However, safety concerns with respect to the adverse effects of the cell-based approach is always controversial (Sukpaita et al., 2019). These cell-based approaches are gaining attention in the field of dentistry. The human pulp stem cells, Dental pulp Stem cells (PDSCs), Stem cells from Apical Papilla (SCAP), Stem cells from exfoliated deciduous teeth (SHED), and human periodontal ligament cells (HPLCs) have high multi-differential potential and are giving promising results in regenerative dentistry. Besides these, another cell fraction has been identified from periapical granulation tissues and are termed as “human periapical cyst-mesenchymal stem cells (hPCy-MSCs)” and suffice with high proliferation and multi-differentiation capacity (Tatullo et al., 2017; Liao et al., 2020). Periodontal regeneration with human periodontal ligament cells (HPLCs) has resulted in excellent clinical success. Clinical trials have reported that HPLCs when seeded on chitosan scaffolds enhance osteogenesis without any toxic effects. It is said that the above-mentioned cells along with chitosan tend to increase the gene expression of osteoblastic cells (RUNX2, ALP, OPN) resulting in enhanced osteogenic potential (Li Y. et al., 2019). Recently in a study by Liao et al., Mesoporous hydroxyapatite/chitosan scaffold recombined with human amelogenenin promoted the proliferation of HPLCs leading to formation of cementum and bone (Liao et al., 2020). Studies have revealed that Stem cells from human exfoliated teeth (SHED) with TGF-β1 when inoculated on chitosan scaffolds show osteogenic potential (Li Y. et al., 2019). Chitosan laden scaffolds and sponges are considered as good delivery vehicles for periodontal regeneration (Su et al., 2014; Vining and Mooney, 2017).

Understanding the mechano-biology of stem cells, a sterile artificial environment which fulfills all the requisites of tissue engineering including the biochemical and mechanical forces should be designed. Stem cells respond to intracellular and extracellular forces. The physiological environment can be altered to regulate the stem cell behavior to achieve their beneficial effects. Mechanical cues like stiffness and viscoelasticity of the scaffold on which the cells are seeded, can regulate the organogenesis and the cell fate *ex-vivo* (Thomas et al., 2017). Modifying the viscoelasticity and stiffness of the chitosan-hyaluronic acid hydrogel as connective tissue tends to promote the proliferation of chondrocytes and the gene expression of ECM markers (Jagodzinski et al., 2004; Brindley et al., 2011).

Mechano-regulation of stem cells through gene expression, proliferation, differentiation forms the basis for osseous regeneration (Lovecchio et al., 2019). It is believed that shear and compressive forces enhance the proliferation of human mesenchymal cells into osteoblasts and produce extracellular matrix. Human bone marrow stromal cells (hBMSCs) due to cyclic loading tend to enhance collagen–I fiber, AL and OC levels leading to osteogenesis (Choi et al., 2018). Tissue engineering models are conducted on 3-D scaffolds to create a physiochemical environment for stem cells. It has been evaluated that cell proliferation, and differentiation and ECM matrix deposition occurs when hBMSCs cultured on chitosan-graphene 3-D scaffold undergo mechano-stimulation (Choi et al., 2018).

#### 2.3.11 Chitosan in medical imaging

Targeted tumor therapy using chitosan nanoparticles permit exceptional prospects beyond standard cancer therapies. Combining such nanoparticles with diagnostic test such as computed tomography, magnetic resonance imaging, and ultrasound imaging, or multi-modal imaging compounds aids easy cancer detection (Sun et al., 2014). Chitosan nanoparticles with multiple contrast agents have been tested for multimodal imaging procedures to counteract disadvantages of single imaging modality. A multimodal approach provides both *in-vitro* and *in-vivo* results effectively. Sun et al. reported that solubility of glycol chitosan when treated with hydrophilic ethylene glycol or PEGylation made it suitable for tumor imaging (Zhang et al., 2013). Besides, chitosan nanoparticles show passive targeting due to higher permeability and retention in cancerous lesions for prolonged duration (Min et al., 2015). This property is also known as enhanced permeability and retention effect.

The traditional method for synthesizing gold nanoparticles lacks stability due to presence of sodium citrate salt. Hence, Sun et al., modified the surface of the gold nanoparticles by eliminating the salt and including a reducing agent, glycol chitosan, which acts as a stabilizer biologically. Also, glycol chitosan is already known as an effective tumor targeting agent in animal models. Coating success was established after observing the change in the refractive index around the coated nanoparticles, using a UV-visible spectrometer, transmission electron microscopy, and Fourier transform infrared spectroscopy. The targeted accumulation in the tumor and its bio-distribution was assessed *in-vivo* using computed tomography (CT) in mice induced with colon cancer. The study results showed enhanced stability of the nanoparticles biologically due to a stabilizing surface coat of chitosan. Moreover, high contrast images of tumor were acquired from the mice by using the coated nanoparticles as a CT contrast agent. The images matched the results of cellular uptake and were highly sensitive to metastatic zones. A selective uptake of the coated nanoparticles was observed in the colon cancer cells compared to macrophages. Thus, the characteristics accumulation of the glycol chitosan coated nanoparticles in cancerous cells demonstrates its efficient tumor targeting capacity which promotes imaging. The authors observed that the ample amine groups of glycol chitosan coated gold nanoparticles serves as zones for chemical conjugation for chemotherapeutic agents and enhances stability to support cancer imaging (Sun et al., 2014).

Zhang et al., studied the efficacy of gadolinium loaded chitosan nanoparticles as magnetic resonance imaging (MRI) contrast agents to target cancerous tissues by enhanced permeability and retention effect. To counteract the shortcomings of gadolinium based chelates such as easy renal filtration and poor contrast, gadolinium was chemically conjugated using ionic gelation with chitosan to prevent its early release and achieve improved retention for extended imaging time. *In-vitro* MRI revealed comparatively high relaxation time of gadolinium loaded chitosan nanoparticles because of surface modification demonstrating its capability as an effective contrast agent. Compared to another commercial contrast agent Magnevist^®^ (same amount of gadonilium without chitosan coating), gadolinium loaded chitosan nanoparticles presented advanced imaging capacity and a high sensitivity aiding early diagnosis. During *in-vivo* MRI, gadolinium loaded chitosan nanoparticles showed higher brightness and retention time compared to Magnevist^®^, prolonging the imaging time considerably. This improvement was attributed to chitosan conjugation with gadolinium. The authors concluded that chemically conjugated gadolinium loaded chitosan nanoparticles holds vast possibility as MRI contrast agent (Zhang et al., 2013).

Min et al., developed an echogenic glycol chitosan-based nanoparticles for ultrasound-based imaging of malignant lesions. The authors used a chemotherapeutic bio-inert agent named perfluoropentane (PFP) which served as an ultrasound gas precursor. The components were turned into glycol chitosan nanoparticles using an oil-water emulsion approach, bearing an anti-cancer drug (docetaxel or doxorubicin)/PFP inner core coated with hydrophilic glycol chitosan. The authors demonstrated that the hydrophobic inner core was essential to stabilize the glycol chitosan coating. The ultrasound imaging capacity of echogenic particles were confirmed by injecting them intravenously in cancer induced mice. Within a minute of injection strong bright images were detected via ultrasound imaging due to effective accumulation of the particles in the cancerous cells. PFP gas production within the nanoparticles helped in retaining the ultrasound signals for an hour. The accumulation was noticed until 2 days after the injection because of due to the enhanced permeability and retention effect produced by promising size of the coated nanoparticles. The ultrasound treated samples displayed 4–7 times increased accumulation and wider distribution of chitosan coated nanoparticles in excised growths. This represents the thorough penetration of chitosan coated nanoparticles into the major vessels and its effective spread to the cancerous tissues. Based on their experimental observations the authors concluded that the echogenic glycol chitosan coated nanoparticles may be tried as ultrasound contrast enhancers in cancer imaging (Min et al., 2015).

Choi et al., developed iodine based echogenic diatrizoic acid-conjugated glycol chitosan nanoparticles as multimodal contrast agents for computed tomography-ultrasound dual imaging. Glycol chitosan-diatrizoic acid compound was formulated chemical conjugating with the amine groups on chitosan. Oil-water emulsion technique was used to introduced iodinated nanoparticles. The resultant contrast agents were directly inserted into cancerous tissues. Notably, the iodide-based echogenic glycol chitosan nanoparticles showed significant accumulation in cancerous tissues. Clear signals were obtained on injecting the contrast agents into tumor sites using both computed tomography and ultrasound imaging modalities. The authors considered the developed agent to perform effectively during a multi-modal imaging approach (Choi et al., 2018).

With all the clinical trial data challenge remains to replicate the same responses in human subjects with tumors and other cancer drug interactions. It is necessary to observe the immune responses and related toxicities. Genetic mutations and varied vascularity of cancerous tissues may generate dissimilar response compared to lab animals. If all such limitations are taken care of then chitosan would have a promising future in targeted cancer imaging and drug therapy.

#### 2.3.12 Chitosan in oral pathology and oncology

**FIGURE 8:**
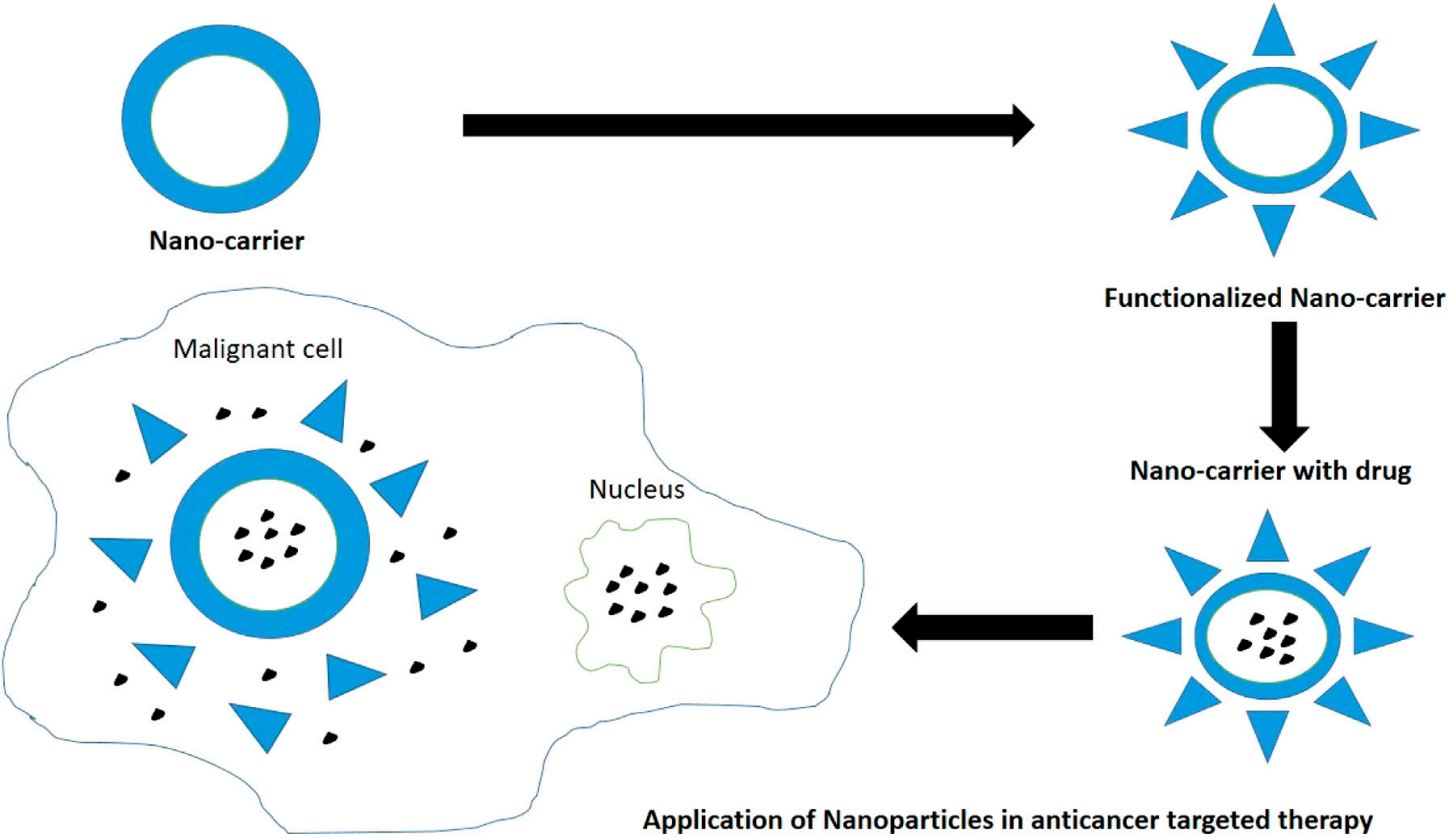
Nanoparticles as a drug-carriers in targeted therapy for treatment of cancers.

Chitosan modified nanocarrier systems can be used as a potential vehicle to target anti-cancer drugs to the various tumors and cause tumor apoptosis as per the individual efficacy of loaded drugs (Figure 8). Surface modifications of chitosan nanoparticles have been postulated which aim to enhance the tumor targeting ability via different mechanisms like receptor or carrier mediated transcytosis. Chitosan presents significant biocompatibility and promotes wound healing at molecular and cell level. Chitosan also acts as a bio-adhesive or mucoadhesive and hydrates the underlying tissues to effectively heal ulcers and relieve pain. It repairs tissues, contracts wound by acting as hemostat, secretes inflammatory mediators, and induces macrophage actions by providing a non-proteinaceous matrix for 3D-tissue growth. Chitosan serves as collagen depositor by releasing N-acetyl-D-glucosamine on depolymerization to promote fibroblast formation. Chitosan modifies bacterial surface morphology, improves cell permeability, causes intracellular constituents’ seepage, and prevents nutrient transport. Hence prevents erythema and secondary infections related to cancer therapy. Chitosan also presents fungistatic property by improving permeability of yeast cells (Mahima et al., 2015).

Min et al., developed an echogenic glycol chitosan-based nanoparticles for ultrasound-based drug delivery to areas of induced squamous cell carcinoma (SCC7 cell lines). An echogenic approach is known to enhance the drug release because of external ultrasound and result in a targeted burst release of the therapeutic agents to tumor sites. In the study the inner core behaved as an efficient reservoir for both the anti-cancer agents and PFPs. The gas precursor successfully retarded gas expansion and maintained stability. The authors demonstrated that the chitosan coated nanosized echogenic particles persisted in the bloodstream for longer periods promoting targeted delivery. Moreover, the outer glycol chitosan coating improved the overall physiochemical characteristics and targeted delivery (Min et al., 2015).

Wimardhani et al., studied the influence of low-molecular-weight chitosan on Ca9-22 cells derived from gingival carcinoma. The study was based on the premise that the low-molecular-weight chitosan showed anticancer effects and were relatively less toxic to non-cancerous cells. Cytotoxic effects were observed with low-molecular-weight chitosan on Ca9-22 cells leading to cell cycle arrest, upsurge in apoptotic DNA fragmentation, and subtle elevation in caspase expressions. The author concluded that the short-term exposure to low-molecular-weight chitosan has encouraging application as an anti-cancer agent (Wimardhani et al., 2014b).

Muthukrishnan et al., studied the effect of chitosan with zinc sulphate for treating and preventing oral mucositis as a side effect of radiotherapy. A WHO mucositis scale was used to grade oral mucositis three times a week. Although during early treatment days no significant changes were observed, around the sixth week a reduced severity was noticed. Pain levels and dysphagia were also reduced. The authors concluded that chitosan-zinc sulphate combination lowered the mucositis severity and aids healing (Muthukrishnan and Shanmughapriya, 2017).

Mahima V.G., examined the influence of 1% chitosan mouthwash on oral mucositis on 20 patients developed after radio-chemotherapy. The study showed significant results in lowering symptoms of oral mucositis such as pain, erythema, ulcerations, and other associated side effects. Chitosan not only demonstrated enhanced healing but also acted as a promising occlusive dressing to alleviate pain and ulcer discomfort due to bio-adhesive properties. No secondary infections and adverse biocompatibility issues were reported during the tests reinforcing chitosan’s ability to work effectively as solution to side effects of radio-chemotherapy. The authors concluded that chitosan is far more efficient than chlorhexidine in alleviating symptoms of oral mucositis (Mahima et al., 2015).

Pornpitchanarong et al., investigated a muco-adhesive based on catechol-modified chitosan/hyaluronic acid nanoparticles as a carrier of doxorubicin for oral cancer. Chitosan and catechol functionalized drug delivery were already reported to boost muco-adhesion individually. Muco-adhesion provides prolonged retention with sustained release of the agents. The authors demonstrated that a significant portion of drug could be loaded into the nanoparticles and sustained release was attainable. A better muco-adhesive property was observed. The combination also induced effective apoptosis of HN22 human oral cancer cells. The authors concluded the said combination as a potential drug carrier for doxorubicin to prevent localized oral cancers (Pornpitchanarong et al., 2020). Figure 7 depicts the usage of nanoparticles as drug carriers in targeted therapy for treatment of various cancers (Sharifi-Rad et al., 2021).

Zhu et al. also reported that a fluorinated chitosan-chlorin e6 (FC-Ce6) nanocarrier for intracellular delivery catalase enhanced photodynamic therapy of oral cancer. They established that fluorine conjugated chitosan nanoparticles exhibited superior anti-cancer activity in contrast to free Ce6 and non-fluorinated CS-Ce6/catalase nanoparticles. Chen et al., tested chitosan nanoparticles encapsulated with 5-aminolevulinic acid (a photo-sensitizer), and IR780 (a near-infrared fluorescence dye used as a photo-thermal agent). However, a single approach using 5-aminolevulinic acid and IR780 had many drawbacks such as tumor recurrence, hydrophilicity of 5-aminolevulinic acid, and low specificity. Chitosan served as a nano-carrier with high biocompatibility and cell membrane permeability. Hence, a combination of photo-thermal and photodynamic agents for a non-invasive oral cancer treatment was developed in the study. The author demonstrated that the said combination displayed improved accumulation in cancerous tissues and showed fluorescence during imaging. Improved photo-thermally augmented photodynamic result for tumor excision was reported with no apparent toxicity. The authors concluded that the combination was safe for use as non-invasive oral cancer therapy (Zhu et al., 2021). Chen et al. formulated a new photothermally enhanced photodynamic therapy platform based on orally administered Chitosan Nanoparticles. Improved photodynamic cytotoxicity to cancer cells was seen with a combination of PTT (photothermal therapy) and PDT (photodyanamic therapy) when compared with photodynamic therapy alone. Additionally, 5-ALA (5-aminolevulinic acid) &IR780 coated Chitosan Nanoparticles exhibited high tumor accumulation and greater ability to fluorescently image tumor tissue (Chen et al., 2020).

Takeuchi et al., designed a sustained release film loaded with rebamipide and chitosan for treating side effects of oral mucositis after chemotherapy. Rebamipide was enforced as a gargle to counter side effects of oral mucositis but its effects were short lived. Chitosan was reinforced into rebamipide for its muco-adhesive and antibacterial characteristics. Along with chitosan, pluronic was added as adhesion enhancer and hydroxyl-propyl methylcellulose served as film former. The release behavior was studied. The authors reported that chitosan caused suppression in the release of rebamipide for nearly half an hour. Whereas, hydroxyl-propyl methylcellulose helped in sustained release and maintained the films shape. The authors concluded that the combination can be effectively used as base for sustained drug delivery. However, additional clinical trials are warranted (Takeuchi et al., 2019)**.**

## 3 Conclusion

Chitosan shows a notable range of properties which makes it valuable for sustainable development due to it being plentiful, decomposable, eco-friendly, and adaptable. Chitosan production has improved in terms of green chemistry due to the harmful chemicals being replaced by solvents with minimum melting points (Eutectic). This has also lead to an overall decrease in energy consumption. An important reason for using chitosan is the presence of a large number of organic groups (hydroxyl and amino groups) in its structure which makes it amenable to chemical modifications (Maliki et al., 2022). This versatility of chitosan makes it especially remarkable for the preparation of suspensions, composites, functionalized materials, or (nano)hybrids for diverse environment-friendly usage and in industrial and health related applications. Chitosan-based nanocomposites, hydrogels, and membranes are being used in regenerative medicine and dentistry. In field of dentistry, it has gained enormous popularity due to its natural existence and biocompatibility and decreased cytotoxicity. However, it still presents certain limitations with regard to its structure and molecular weight. Nonetheless, this natural nanomaterial is being extensively used and has an excellent potential to stretch out its biotic properties in the near future. There is hardly any clinical evidence of chitosan-based derivatives in the field of dentistry and more clinical data should be added on. Being the second abundant biopolymer in nature after cellulose, the potential of chitosan as sustainable future material in dentistry and medical needs further exploration. Continuous investigation into nanobiotechnology related to computer science ought to be carried out to enhance the present state of medicine and create pharmaceuticals with strong therapeutic efficacy to reduce patients’ discomfort while optimizing the effectiveness of therapeutic agents.
